# Vertical sleeve gastrectomy induces distinctive transcriptomic responses in liver, fat and muscle

**DOI:** 10.1038/s41598-021-81866-5

**Published:** 2021-01-27

**Authors:** Chang Ho Ahn, Eun Hye Choi, Hyunjung Lee, Woochan Lee, Jong-Il Kim, Young Min Cho

**Affiliations:** 1 Department of Internal Medicine, Seoul National University College of Medicine, 101 Daehak-ro, Jongno-gu, Seoul, 03080 Republic of Korea; 2 Department of Internal Medicine, Seoul National University Bundang Hospital, Seongnam, Republic of Korea; 3 Department of Internal Medicine, Seoul National University Hospital, Seoul, Republic of Korea; 4 Department of Biomedical Sciences, Seoul National University, Seoul, Republic of Korea; 5 Medical Research Center, Genomic Medicine Institute, Seoul National University, Seoul, Republic of Korea

**Keywords:** Diabetes, Obesity

## Abstract

Vertical sleeve gastrectomy (VSG) is the most commonly performed bariatric/metabolic surgery, exhibiting a high rate of diabetes remission in humans. To elucidate the molecular mechanisms of VSG, we performed transcriptomic analysis of the liver, fat, and muscle in VSG mice. C57BL/6 mice fed a high-fat diet were randomly assigned to sham or VSG surgery. The sham-operated mice were fed ad libitum (sham group) or pair-fed (sham-PF group) matching their food intake to the VSG-operated mice. Comparative transcriptomic analysis of the liver, fat, and muscle using RNA sequencing was performed. VSG reduced body weight and improved glucose tolerance compared to the sham group, but not more than the sham-PF group. Improvement in fatty liver and adipose tissue inflammation was comparable between VSG and sham-PF. However, global gene expression profiles showed distinctive changes in the liver, fat, and muscle of the VSG group compared to both the sham or sham-PF groups. The liver showed the most prominent gene expression changes. Immune response-related pathways were commonly upregulated in the three organs of the VSG group compared to the sham or sham-PF. VSG induces organ-specific gene expression changes in the liver, fat, and muscle, which may play critical roles in metabolic improvements after VSG.

## Introduction

Bariatric surgery is currently the most effective and durable treatment for obesity. Furthermore, its profound metabolic effects including remission of type 2 diabetes mellitus (T2DM) introduced a new concept that is called ‘metabolic surgery’^1^. Among various types of bariatric/metabolic surgery, vertical sleeve gastrectomy (VSG) is the most popular procedure worldwide^2,3^. VSG removes approximately 80% of the stomach along the side of the greater curvature, leaving a tube-like remnant stomach. In this regard, VSG was initially considered a restrictive surgery^4^. However, a growing body of evidence suggests that the restriction of food intake is not the primary mechanism driving metabolic improvements after VSG^5^.

VSG has pleiotropic effects on whole-body metabolism and its regulation^6^. VSG decreases hedonic food intake. In obese patients, the hedonic rating for high-fat food was decreased after VSG^7^. Bile acid pool is another component affected by VSG. In mice, total circulating bile acid level was increased after VSG^8^. Specifically, conjugated bile acid levels were increased, but unconjugated bile acid levels were decreased^8^. Bile acids not only serve critical roles in fat absorption, but they also act through cellular receptors including FXR and TGR5. FXR knock-out mice showed diminished effects of VSG, suggesting a pivotal role of FXR to mediate the effects of VSG^9^. TGR5 knock-out mice showed reduced, but nevertheless significant improvements of glucose metabolism after VSG^8,10^. Bariatric surgery also affects the secretion of gut hormones^11^. After VSG, postprandial secretion of glucagon-like peptide-1 (GLP-1) and peptide YY (PYY) was increased^12^. The increase of GLP-1 secretion was a significant predictor of diabetes remission after surgery^12^. The gut microbial composition is changed after VSG. The *Bacteroides* genus was decreased after VSG, which was associated with low adiposity and improved glucose metabolism^13^.

In addition to the abovementioned effects of VSG, previous studies showed distinctive effects of bariatric surgery on major glucose-metabolizing organs, liver, fat and muscle. In the liver, hepatic glucose production and fat content were decreased as early as 1 week after bariatric surgery in obese patients^14^. VSG-induced gene expression changes of the liver were independent of weight loss, where genes involved in bile acid metabolism were mainly changed^15,16^. In adipose tissue, bariatric surgery induced decreased lipolysis and tissue inflammation^17,18^. In gene expression analyses, pathways involving branched chain amino acid, NAD^+^, and glutathione metabolism were upregulated in both visceral and subcutaneous adipose tissue 1 year after bariatric surgery in obese patients^19^. The muscle showed improved lipid metabolism and mitochondrial function 1 year after bariatric surgery, which was accompanied by various epigenetic changes^20^. To systematically explore the effects of VSG on major glucose-metabolizing organs (i.e., the liver, fat and muscle), we analyzed global gene expression of the three organs in VSG-operated mice.

## Materials and methods

### Animals

Six-week-old male C57BL/6 N mice were purchased from Orient Bio, Seongnam, Korea. After 1 week of acclimation, mice were fed 60% high-fat diet (D12492, Research Diets, New Brunswick, NJ, USA) for 12 weeks, and randomly divided into 3 body weight-matched groups: sham, sham-pair feeding (sham-PF), and VSG groups. The sham-PF group was matched to the VSG group one-by-one and given the same amount of high-fat diet eaten by the VSG group. The mice were housed 3 or 4 mice per cage and then individually from 8 weeks before surgery. The housing environment was maintained in a controlled temperature (25 °C) and 12-h light/dark cycle (light off at 20:00 h). All procedures for the animal study were approved by the Institutional Animal Care and Use Committee of Seoul National University Hospital (approval no. 15-0113-C1A1) and were conducted in accordance with the National Institutes of Health Guide for the Care and Use of Laboratory Animals guideline for the ethical treatment of animals. All animal studies were carried out in compliance with the ARRIVE guidelines^21^.

### Surgical procedure and perioperative care

Mice were fasted for 18 h before surgery. The surgical area of the abdomen was depilated using thioglycolic acid cream (Niclean, Ildong Pharmaceuticals, Seoul, Korea). After induction of isoflurane anesthesia, the abdomen was disinfected with povidone-iodine solution and midline laparotomy was performed. The stomach was gently exposed and the spleen was separated from the stomach. The lateral stomach was excised along with a virtual line from gastroesophageal junction to pylorus-duodenum junction leaving a tubular remnant of the stomach. The remaining stomach was approximated and closed by a running suture with 6-0 Vicryl. If leakage was suspected by visual inspection, we added additional sutures. Thereafter, the intraperitoneal cavity was washed with warm saline 3 or 4 times. Dry gauze was gently applied to remove any remaining fluid in the cavity. The abdominal fascia and the skin were closed by a running suture using 6-0 Vicryl and 6-0 nylon, respectively. Antibiotics (ceftriaxone 50 mg/kg/day) was administered before surgery and until postoperative day 2. Normal saline 20 ml/kg was administered after surgery to prevent dehydration. Analgesics (meloxicam 1 mg/kg/day) were administered after surgery and until postoperative day 2. The sham surgery included midline laparotomy, stomach isolation and closing the incision. The duration of sham operation was matched to that of the VSG group by waiting for 10 to 20 min after stomach isolation while the abdomen was covered by warm saline-soaked gauze. No oral intake was allowed for postoperative 24 h. Water was provided for 1 day followed by 5 days of liquid diet (Nucare, Daesang Welllife, Seoul, Korea). On postoperative day 6, 1 g of high-fat diet was reintroduced with liquid diet. If the mouse looked well and ate the high-fat diet, then ad libitum high-fat diet was given without liquid diet. The body weight and food intake were monitored daily.

### Insulin tolerance test

Mice were fasted for 4 h and given an intraperitoneal injection of insulin (0.75 IU/kg). Blood glucose was measured with a glucometer (AccuCheck, Roche Diagnostics, Indianapolis, IN, USA) from the tip of the tail vein at 0, 15, 30, 60, and 90 min after the insulin injection.

### Glucose tolerance test

The mice underwent both intraperitoneal glucose tolerance test (IPGTT) and oral glucose tolerance test (OGTT) 1 week apart. Mice fasted for 12 h overnight was given 20% dextrose (1 g/kg) by intraperitoneal injection and oral gavage, respectively. Blood glucose was measured with a glucometer (AccuCheck) from the tip of the tail vein at 0, 15, 30, 60, and 120 min after glucose administration.

### Glucose-stimulated insulin and GLP-1 secretion in vivo

On a separate day at least 1 week apart from any blood measurement, mice fasted for 12 h overnight were given 20% dextrose (1 g/kg) by oral gavage. Before and 15 min after the oral gavage, approximately 100 μl of blood was obtained. Blood was cold-centrifuged and plasma was stored at − 80 °C until the measurement of insulin and total GLP-1. Plasma insulin and total GLP-1 level were measured by ELISA (#90080 and #81508, respectively, CrystalChem, Downers Grove, IL).

### Tissue collection and RNA sequencing

After euthanasia, the liver, epididymal fat and soleus muscle were isolated from the sham, sham-PF, and VSG groups. Tissues were stored in RNA*later* solution (Invitrogen, Carlsbad, CA, USA) at − 20 °C. Total RNA was extracted using RNeasy Plus Universal kits (Qiagen, Valencia, CA, USA). RNA quality was checked based on RNA integrity number (RIN). All samples had RIN over 9.0. cDNA library was constructed with the TruSeq RNA library kit using 1 μg of extracted RNA. The protocol consisted of polyA-selected RNA extraction, RNA fragmentation, random hexamer primed reverse transcription and 100 nt paired-end sequencing by Illumina HiSeq4000 (Illumina Inc., San Diego, CA, USA). Sequence reads were aligned to the mouse reference genome (mm10) and gene expression values were calculated from aligned reads using RSEM-1.2.31. Differentially expressed genes (DEGs) were determined using DESeq2 package in three comparisons: sham-PF versus sham, VSG versus sham, and VSG versus sham-PF^22^. The criteria for DEG was adjusted *P* value < 0.05 and absolute fold change ≥ 1.5. Enrichment analysis of gene ontology biologic process and KEGG pathway^23^ was done using DAVID software^24^. The enrichment analysis was done for upregulated genes and downregulated genes separately and the genes with raw *P* value < 0.05 were used for the analysis.

To compare the gene expression profiles with Roux-en-Y gastric bypass (RYGB) mouse model, the Gene Expression Omnibus (GEO) database (https://www.ncbi.nlm.nih.gov/gds/) was searched for datasets that included the gene expression profiles of the liver, fat, and muscle samples collected from mouse RYGB models. GSE113823 is the dataset deposited from a study that investigated the gene expression of different organs after RYGB^25^. In that study, the gene expression profiles of the liver, inguinal fat, and gastrocnemius muscle were analyzed and compared with weight-matched sham control group. The dataset of postoperative 9 weeks was used. We downloaded the FASTQ files and processed it using the same protocols of our current study.

### Flow cytometry analysis of the stromal vascular fraction

The dissected epididymal fat samples were minced in DMEM with 1 mg/ml collagenase P (Roche, Mannheim, Germany) and 5% BSA. After 45 min of shaking incubation at 37 °C, any debris was filtered using nylon mesh. The stromal vascular fraction (SVF) was separated via centrifugation (500 g for 10 min). The SVF was washed and incubated with antibodies for further flow cytometry analysis. For the analysis of macrophage population, the SVF was stained with following antibodies: CD11b-FITC (BD 557396, BD Biosciences, Franklin Lakes, NJ), F4/80-Bv421 (BD 565411), CD206-APC (BD 565250), and MHC II-PE (BD 562010). For the analysis of lymphocyte population, the SVF was stained with following antibodies: CD19-APC-Cy7 (BD 561043) and CD3-PE-Cy5 (BD 561108). Samples were analyzed using a BD FACSCanto and FACSDiva software (BD Biosciences, Franklin Lakes, NJ). Data were processed with FlowJo software (Tree Star Inc. Ashland, OR).

### Histologic analysis

The tissues were fixed in 4% paraformaldehyde solution and embedded in paraffin. The tissue sections were stained with hematoxylin and eosin (H&E). Nonalcoholic fatty liver disease severity score which estimates the severity of fatty liver by the summation of steatosis, lobular inflammation and hepatocyte balloon degeneration was calculated in a blinded fashion. The lipid droplet areas of the H&E stained liver were quantified using ImageJ^26^ according to a previous protocol^27^. The size of adipocyte was measured using ImageJ. The number of crown-like structure was calculated per 5 high power field (× 200) to estimate the adipose tissue inflammation.

### Statistical analysis

Data in the graph were expressed as the mean ± SEM, unless otherwise indicated. The area under the curve (AUC) was calculated using the trapezoidal rule. Time-series data were analyzed using repeated measures ANOVA followed by Sidak’s post hoc test. Three group comparisons were performed using one-way ANOVA followed by Tukey’s post hoc test. Pearson correlation of Log_2_ fold change of the gene expression was used to determine similarity between the gene expression data of VSG and RYGB model. Data were analyzed using Prism v8.0 (GraphPad, San Diego, CA, USA) and R version 3.5.0 (R Foundation for Statistical Computing, Vienna, Austria). *P* value < 0.05 was considered statistically significant. The statistical analysis used for RNA sequencing was described in the previous section.

## Results

### Body weight and glucose tolerance

**Figure 1:**
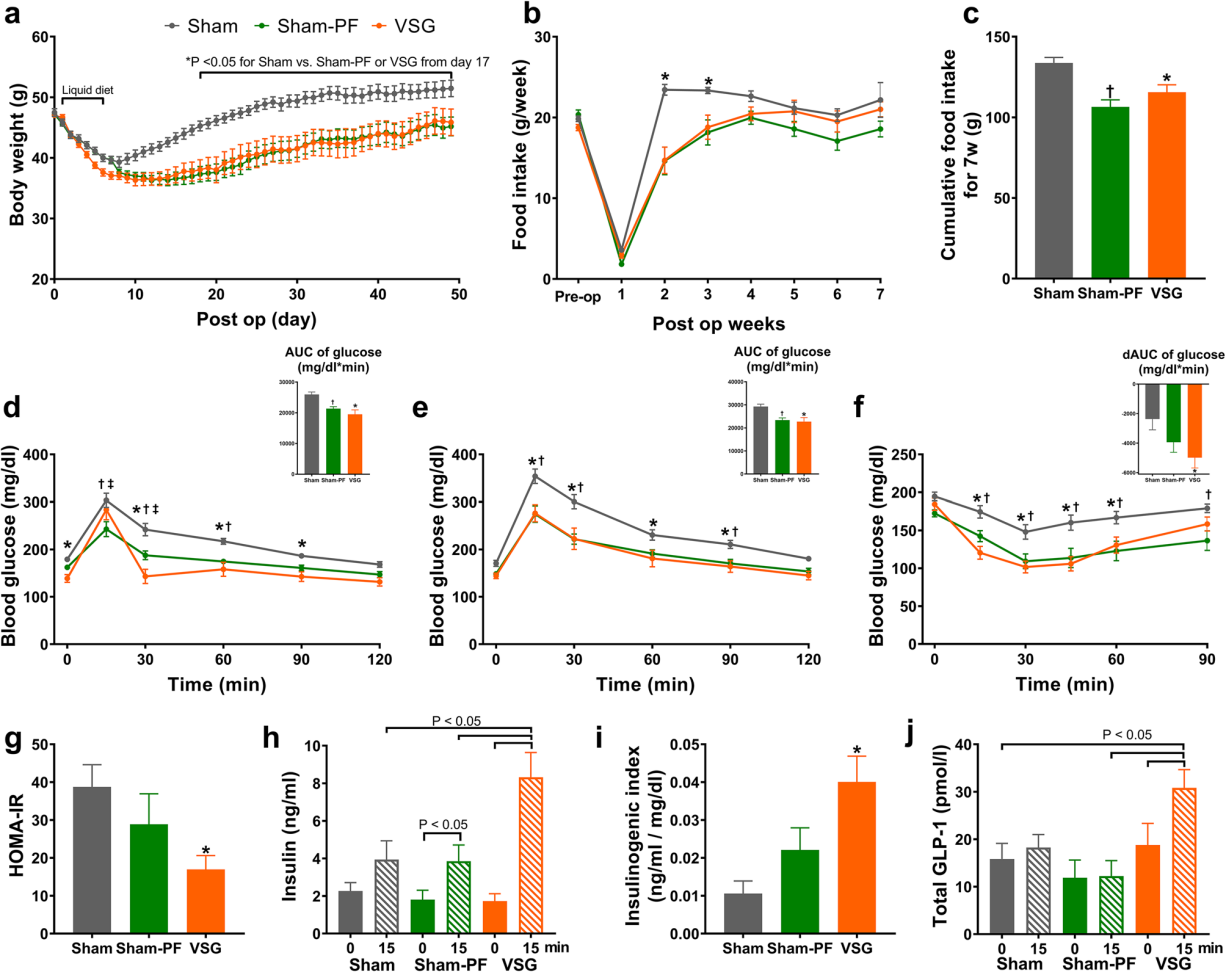
VSG induced sustained weight loss and improved glucose tolerance. (**a**) Body weight, (**b**) weekly food intake and (**c**) cumulative food intake during 7 weeks after surgery. (**d**) Blood glucose levels during the oral glucose tolerance test and the AUC of glucose levels (inset figure). (**e**) Blood glucose levels during the intraperitoneal glucose tolerance test and the AUC of glucose levels (inset figure). (**f**) The blood glucose levels during the insulin tolerance test and the decremental AUC, which is the area under the curve of the glucose levels below the baseline glucose level (inset figure). (**g**) HOMA-IR is defined as fasting glucose level (mg/dl) × fasting insulin level (μU/ml)/405. (**h**) The plasma insulin levels of fasted and 15 min after the oral glucose challenge. (**i**) Insulinogenic index is the increment of the insulin divided by the increment of the glucose level between fasting and 15 min after the oral glucose challenge. (**j**) The plasma GLP-1 levels of fasted and 15 min after the oral glucose challenge. N = 12 in each group. Comparison: Repeated measured ANOVA with Sidak’s post hoc test. *P < 0.05 for sham versus VSG. Data are mean with SEM. ^†^*P* < 0.05 for sham versus sham-PF. ^‡^*P* < 0.05 for sham-PF versus VSG.

All three mice groups lost significant amounts of body weight during the early postoperative period (Fig. 1a). The VSG group induced slightly more body weight loss than the sham group during the liquid diet period and exhibited lower body weight regain thereafter. This resulted in significant weight differences between the VSG and the sham group and the difference was maintained during the rest of the study period. The body weight of the sham-PF group was initially paralleled with that of the sham group during the liquid diet period. Then, the trajectory of the body weight of the sham-PF group followed that of the VSG group after the initiation of pair feeding of the high-fat diet. The food intake was well matched between the VSG and the sham-PF group (Fig. 1b). However, during postoperative 6 and 7 week, the sham-PF group consumed slightly lower amount of food than the VSG group. Compared with the sham group, the VSG group consumed less food only during the first 3 weeks after the surgery (Fig. 1b). The cumulative food intake was similar between the VSG and the sham-PF group, both of which were lower than the sham group (Fig. 1c).

Both the VSG and the sham-PF group demonstrated better glucose tolerance than the sham group during the OGTT and IPGTT (Fig. 1d,e). During the OGTT, the VSG group showed a higher peak of blood glucose at 15 min than the sham-PF group and a steeper decline nearly to the baseline level at 30 min (Fig. 1d). This unique pattern observed during the OGTT was not seen during the IPGTT (Fig. 1e). The AUC of blood glucose levels during the OGTT and IPGTT was similar between the VSG and the sham-PF groups, both of which were lower than those of the sham group (Fig. 1d,e).

During the insulin tolerance test, the decrement of blood glucose level was significantly greater in the VSG and sham-PF groups than in the sham group (Fig. 1f). Homeostatic model assessment of insulin resistance (HOMA-IR)^28^ was significantly lower in the VSG group than in the sham group; however, the sham-PF showed a nominal decrease compared to the sham group (Fig. 1g).

To measure postprandial insulin and GLP-1 secretion, blood was sampled at baseline and 15 min after an oral glucose load. Postprandial insulin levels were significantly higher in the VSG group than in both the sham and sham-PF groups (Fig. 1h). The insulinogenic index at 15 min (the increment of insulin divided by the increment of glucose in the blood) was significantly higher in the VSG group than in the sham group (Fig. 1i), which also tended to be higher than in the sham-PF group (*P* = 0.070). Postprandial plasma GLP-1 levels were higher in the VSG group than in both the sham and the sham-PF groups (Fig. 1j). Fasting GLP-1 levels were not significantly different among the three groups.

### Histologic analysis

**Figure 2:**
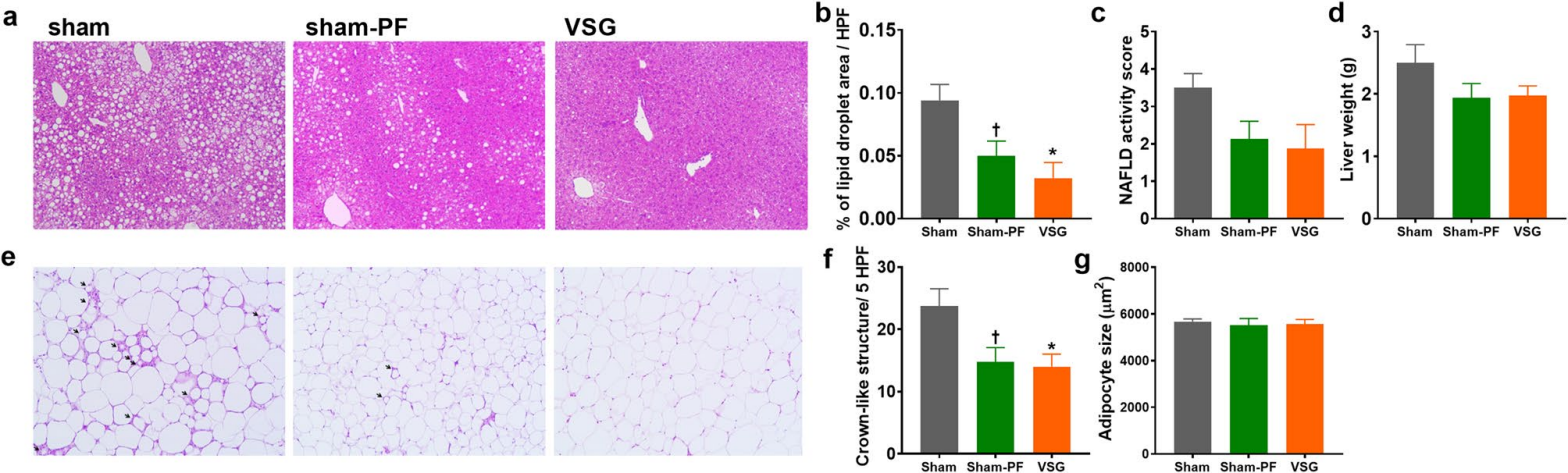
Histologic evaluation of the liver and fat. (**a**) The representative images of the H&E staining of the liver tissue. (**b**) The lipid droplet area of the liver, (**c**) NAFLD activity score and (**d**) liver weight. (**e**) The representative images of the H&E staining of the fat tissue. Arrows indicate crown-like structures. (**f**) The number of crown-like structure per high power field and (**g**) adipocyte size. N = 8 in each group. Comparison: ANOVA with Tukey’s post hoc test. *P < 0.05 for sham versus VSG. ^†^*P* < 0.05 for sham versus sham-PF.

On the histologic examination of the liver, fatty liver was apparently improved in the sham-PF and VSG groups compared to the sham group (Fig. 2a). The lipid droplet area was significantly lower in the VSG and sham-PF groups than the sham group (Fig. 2b). Nonalcoholic fatty liver disease activity score of the VSG and sham-PF groups tended to be lower than that of the sham group (*P* = 0.08) (Fig. 2c). The liver weight also tended to be lower in the VSG and sham-PF groups than in the sham group (Fig. 2d).

On the histologic examination of the epididymal fat, the sham-PF and the VSG groups had fewer numbers of crown-like structures compared to the sham group (Fig. 2e,f). The mean adipocyte size was similar among the three groups (Fig. 2g).

### Global gene expression analyses

**Figure 3:**
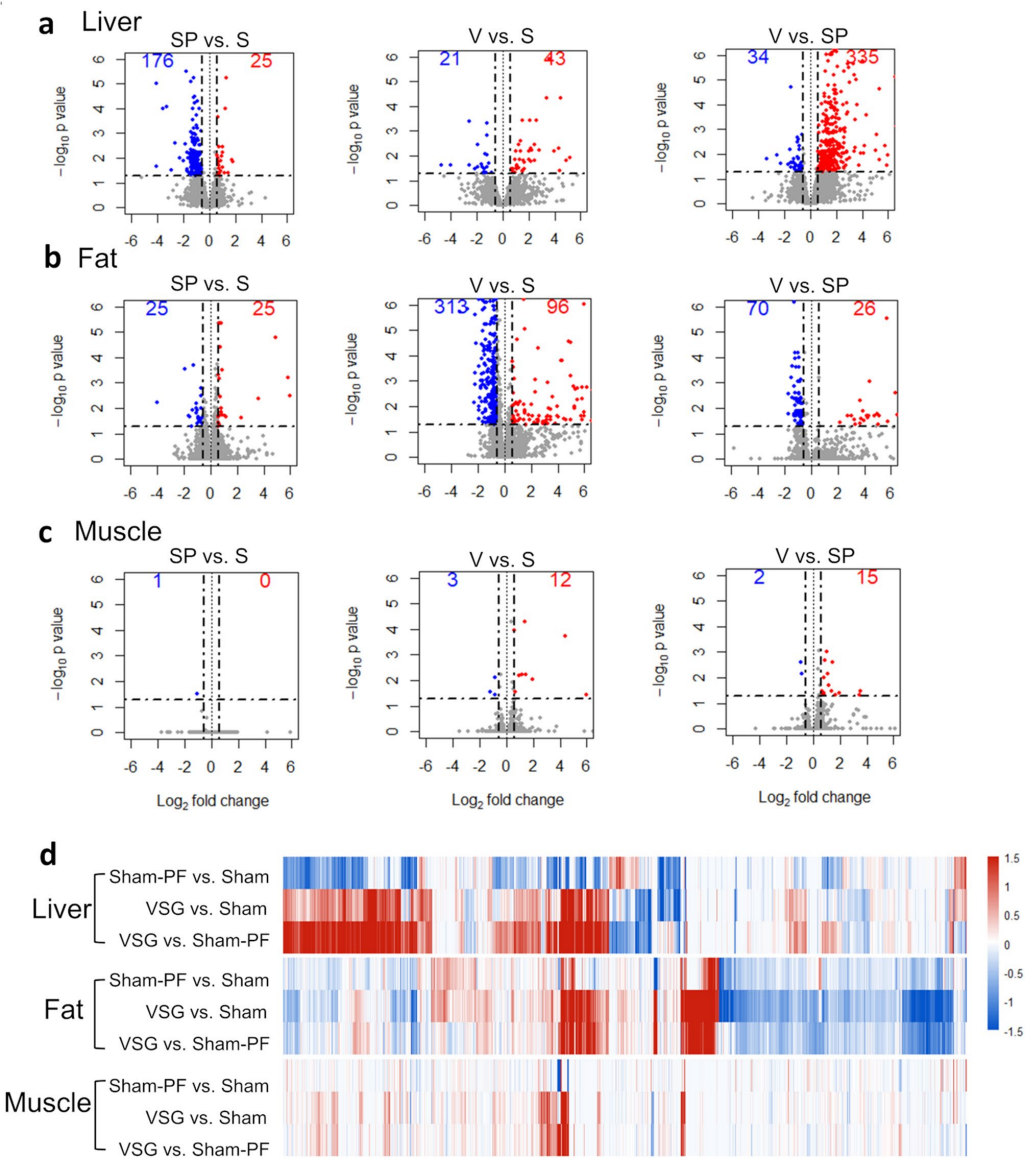
Global gene expression profiles of the liver, fat and muscle. The volcano plot of (**a**) the liver, (**b**) fat and (**c**) muscle. The DEG was defined as adjusted *P* value < 0.05 and fold change ≥ 1.5. (**d**) Heatmap showing log_2_ fold change in the three comparison: sham-PF versus sham, VSG versus sham, and VSG versus sham-PF. Each column represent a gene and genes that showed adjusted *P* value < 0.05 in any of the three organs are included in the heatmap. The numbers of mice included in the RNA sequencing analysis were 4, 3, and 3 for the sham, sham-PF, and VSG group, respectively. S, Sham group; SP, sham-PF group, V, VSG group.

The global gene expression profile showed a distinctive pattern in the liver, fat, and muscle (Fig. 3a–d). In the liver, the number of DEG was greater in the comparison between VSG versus sham-PF than VSG versus sham or sham-PF versus sham (Fig. 3a). The global gene expression profile of the liver changed in a seemingly opposite direction in the VSG and the sham-PF group compared to the sham group, which suggests that the response to the surgery, which was shown in the VSG group, and the response to the calorie-restriction alone, which was shown in the sham-PF group, appeared to be distinctive (Fig. 3d).

In the fat, the number of DEG was greater in the comparison between VSG versus sham than VSG versus sham-PF or sham-PF versus sham (Fig. 3b). Compared to the sham group, the global gene expression profile of the fat changed in a similar direction in the VSG and the sham-PF group (Fig. 3d). In the muscle, however, the number of DEG was much smaller than in the liver or fat (Fig. 3c). Global gene expression profiles showed only small changes by VSG or calorie restriction (as is shown in the sham-PF group) in the muscle (Fig. 3d).

### VSG versus RYGB

**Figure 4:**
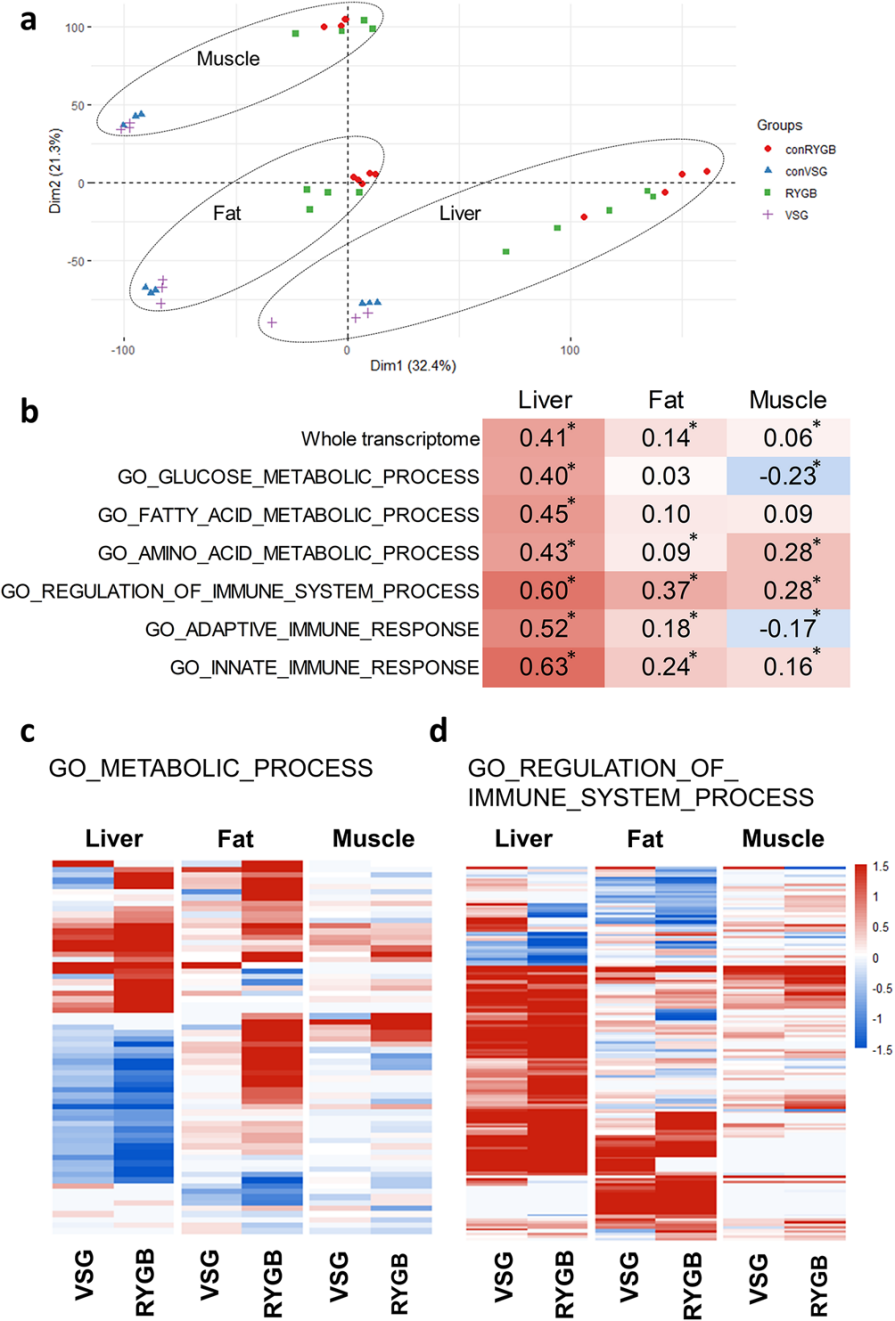
The comparison of gene expression profile between the VSG and RYGB model. (**a**) PCA plot of the VSG and RYGB model and their respective control groups. conRYGB, weight-matched control mice of the RYGB mice; conVSG, pair-fed control mice of the VSG mice. (**b**) Correlation between the gene expression changes of the VSG and RYGB model. Red indicates a positive correlation, while blue indicates a negative correlation. ‘*’ denotes significant by *P* value < 0.05. All other correlations were significant with *P* value < 0.001. The expression patterns of the genes of the (**c**) metabolic process and (**d**) the immune system process in the VSG and RYGB model. Heatmap shows log_2_ fold change. Each row represents a gene. Any genes that showed adjusted *P* value < 0.05 in any of the three organs are included in the heatmap.

RYGB and VSG are the two most popular types of bariatric surgery^2^. They create different anatomical alterations in the gastrointestinal tract and may have different effects on major organs for the glucose homeostasis including the liver, fat, and muscle. To compare the gene expression changes between RYGB and VSG models, we analyzed a GEO dataset by Ben-Zvi et al. that was composed of the liver, fat, and muscle samples from RYGB-operated mice and body weight-matched sham-operated mice^25^. The gene expression changes of the RYGB group versus the weight-matched sham group from the former dataset and of the VSG group versus the sham-PF group from the current study were compared. The global gene expression profile was distinctive between the VSG and RYGB model, which mainly reflects the different experimental settings (Fig. 4a). When we examine the correlation of the fold-changes of the gene expression between VSG and RYGB in an organ-specific manner, the correlation was higher in the liver than the fat or muscle (Fig. 4b). When the correlation of gene expressions involved in each specific pathway was analyzed, genes involved in the immune system process showed a higher correlation between VSG and RYGB than other pathways including metabolism-related pathways (Fig. 4b). The correlation of the gene expression in the glucose and fatty acid metabolism pathway between VSG and RYGB was statistically significant only in the liver (Fig. 4b). The effects of RYGB on the gene expression of the liver, fat, and muscle were in a similar direction as VSG, but with higher fold-changes than VSG for both the metabolism-related genes and the immune response-related genes (Fig. 4c,d).

### Pathway analyses of the gene expression data

**Figure 5:**
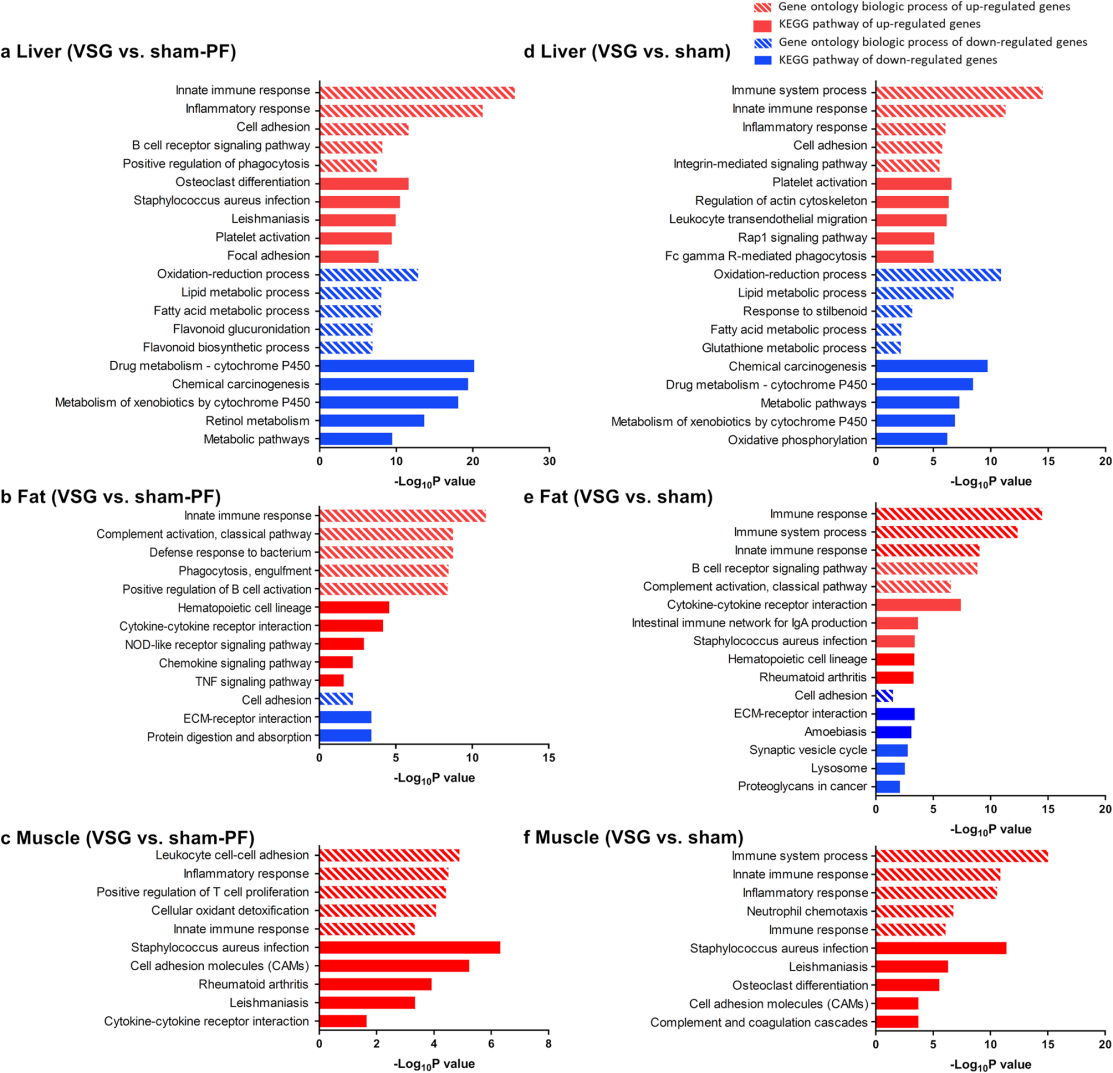
Pathway analyses of the liver, fat and muscle in the VSG group. The enrichment analyses of gene ontology and KEGG pathways in (**a**) the liver, (**b**) fat, and (**c**) muscle in the VSG group compared to the sham-PF group and (**d**) the liver, (**e**) fat, and (**f**) muscle in the VSG group compared to the sham group are summarized. Top 5 gene ontologies or KEGG pathways with adjusted *P* value < 0.05 are shown.

Pathway analyses of the gene ontology and KEGG pathways showed that immune response-related pathways were commonly upregulated in the three organs of the VSG group compared to the sham-PF group (Fig. 5a–c). Specifically, ‘innate immune response’ was significantly enriched in the liver, fat, and muscle. B cell-related pathways were enriched in the liver and fat. At the gene level, *Ifngr1*, which is a receptor of IFN-γ and induces proinflammatory M1 polarization of macrophages was downregulated in the liver, fat, and muscle of the VSG group (Supplementary Table S1). Flow cytometry analysis of the stromal vascular fraction isolated from the epididymal fat showed a tendency of increased anti-inflammatory M2 macrophages (Supplementary Figure S1a–c). *Cxcl13*, a B cell chemoattractant, was also a common DEG in the liver, fat, and muscle of the VSG group (Supplementary Table S1). *Cxcl13* is involved in B cell-related pathways and was upregulated in the three organs. Flow cytometry analysis of the stromal vascular fraction isolated from the epididymal fat showed increased CD19^+^/CD3^+^ lymphocyte ratio, suggesting increased B cell-to-T cell ratio among the lymphocyte population in the VSG group compared to the sham-PF group (Supplementary Figure S1d–f). In the VSG group, compared to the sham group, immune response-related pathways were also commonly upregulated in the liver, fat, and muscle (Fig. 5d–f).

Metabolism-related pathways were enriched in the liver of the VSG group compared to those of the sham-PF group (Fig. 5a). ‘Lipid metabolic process’ and ‘fatty acid metabolic process’ were enriched in the downregulated DEGs of the liver. At the gene level, the expression of metabolism-related genes was also changed mainly in the liver (Supplementary Figure S2a). In the liver of the VSG group, genes involved in the glycolysis pathway, including *Hk1, Hk3,* and *Pkm* were upregulated, while these genes were downregulated in the liver of the sham-PF group compared to the sham group (Supplementary Figure S2b). The genes involved in the fatty acid synthesis and elongation processes including *Acaca*, *Fasn*, and *Elovl*, were downregulated in the liver of the VSG group compared to that of the sham group, which was not seen in the sham-PF group. In the VSG group compared to the sham group, metabolism-related pathways including ‘lipid metabolic pathway’ were also enriched in the downregulated DEGs of the liver (Fig. 5d).

## Discussion

In this study, we focused on the effects of VSG on gene expression in the liver, fat, and muscle, the three major glucose-metabolizing organs. The VSG group exhibited improved glucose tolerance and reduced body weight compared to the sham group. However, the glucose tolerance and body weight of the VSG group were similar to those of the sham-PF group. Although histologic changes were not different between the VSG and the sham-PF group in the liver and fat, the gene expression profile showed distinctive changes, which implies additional mechanisms of VSG on metabolic improvements other than calorie restriction alone.

The global gene expression profile showed a much larger change in the liver compared to the fat and muscle. The muscle showed only small changes in global gene expression. In a previous study^29^, a rat VSG model showed that the significant decrease of hepatic glucose production was the major contributor to the improvement of glucose tolerance than sham or sham-PF control rats. In the same study, glucose clearance by peripheral tissues was not different between the VSG and the sham or the sham-PF group^29^. In a study of obese patients with T2DM, HOMA-IR, which mainly reflects hepatic insulin sensitivity, was improved as early as 3 days after VSG before any significant weight loss occurred^30^. In another study, the hepatic insulin sensitivity measured by a hyperinsulinemic–euglycemic clamp was significantly improved 1 week after RYGB in obese patients, while peripheral insulin sensitivity was unchanged^31^. The liver showed the most prominent gene expression changes in our study, suggesting that the liver is the primary organ mediating the systemic effects of VSG.

Effects of VSG on hepatic glucose metabolism can be summarized as the upregulated glycolytic pathway. Genes involved in the rate limiting and irreversible steps of glycolysis, *Hk1*, *Hk3*, and *Pkm*, were upregulated in the liver of the VSG group compared to that of the sham-PF group. These genes were downregulated in the sham-PF group compared to the sham group. The calorie intake in the sham-PF group was restricted to match to the calorie intake of the VSG group. In the calorie-restricted state, the activity of gluconeogenic enzymes is enhanced and that of glycolytic enzymes is suppressed in the liver^32,33^. VSG might reverse this calorie restriction-induced changes of hepatic glucose metabolism. Although we could not specify the mechanism of how VSG affects hepatic gene expression, the changes of bile acid pool and subsequent FXR signaling could mediate this effect. FXR is a nuclear receptor of bile acids that coordinates hepatic glucose metabolism according to fasting and feeding state^34^. In a murine study, levels of circulating bile acids were increased and the expression of FXR target genes were enhanced in the VSG group^15^. Activation of FXR by a chemical agonist or gene transfer suppressed *Pepck* and *G6pc*, genes involved in gluconeogenesis in the liver^35^. In addition, a previous report that FXR is required for the beneficial effects of VSG on glucose metabolism^9^ supports the possibility that FXR mediates the effects of VSG on hepatic gene expression.

In our VSG model, immune response-related pathways were upregulated in the liver, fat, and muscle. There are other previous studies also reported the increased immune response in peripheral tissues after bariatric surgery. Hagman et al. reported that, at postoperative 1 year after RYGB or VSG, patients exhibited 35 kg weight loss on average, but the number of neutrophils, dendritic cells, macrophages, and T cells were increased in the subcutaneous adipose tissue^36^. Frikke-Schmidt et al. examined the adipose tissue immune cell population in VSG-operated mice^37^. The number of T cells and macrophages were increased in the epididymal fat of the VSG group compared to that of the sham and the sham-PF group. A RYGB mouse study also reported that RYGB induced a stronger immune response in the liver, fat, and muscle characterized by upregulation of *Il33*^25^. However, there are other studies that reported opposite results. The expression levels of inflammatory cytokines including *Ccl2*, *Il6*, and *TNF-α* were decreased in the adipose tissue of the RYGB-operated patients after achieving 20% body weight loss^38^. Systemic levels of IL-6 were decreased in the short- and long-term periods after bariatric surgery^39^. Collectively, these results suggest that VSG might differentially affect each specific immune pathway in different contexts such as different postoperative time points and target organs.

The flow cytometry analysis of the stromal vascular fraction was performed to examine whether the changes in the gene expression was related to the changes in the immune cell population in the adipose tissue. In the VSG group, compared to the sham-PF group, the proportion of M2 macrophage tended to be higher and B cell to T cell ratio was modestly increased. Similarly, it was reported that VSG, compared to pair feeding, increased CD11c^−^ macrophage (an M2 phenotype) in the adipose tissue in high fat diet-fed obese mice^37^. The metabolic role of B cells residing in the adipose tissue is yet to be fully elucidated^40^. It was reported that regulatory B cells attenuated adipose tissue inflammation and had beneficial effects on glucose metabolism, while another B cell subset, B2 subtype, promoted adipose tissue inflammation and insulin resistance^41,42^. Our study showed that VSG altered the immune cell population in the adipose tissue and the immune response gene expression in the liver, fat and muscle, which warrants further mechanistic study to elucidate the role of these immune responses in metabolic improvements following VSG.

In summary, VSG improved glucose tolerance and induced sustained body weight loss to a similar extent as pair-fed sham controls. VSG induced global gene expression changes in the liver, fat, and muscle. Among the three organs, the liver showed the most prominent gene expression changes after VSG. Immune response-related pathways, in particular, were commonly upregulated in the liver, fat, and muscle. VSG-induced gene expression changes in the liver, fat and muscle may play a critical role in the metabolic improvements after VSG.

## Supplementary Information

Supplementary Information

## Data Availability

The datasets generated during and/or analysed during the current study are available from the corresponding author on reasonable request.
