# Supplementary material for: Vertical sleeve gastrectomy induces distinctive transcriptomic responses in liver, fat and muscle
Source: Sci Rep. 2021 Jan 27;11:2310. doi: 10.1038/s41598-021-81866-5 (PMC7840766; doi:10.1038/s41598-021-81866-5)
Supplement: Supplementary file 1 — Supplementary Information [file 41598_2021_81866_MOESM1_ESM.docx]

**Vertical Sleeve Gastrectomy Induces Distinctive Transcriptomic Responses in Liver, Fat and Muscle**

Chang Ho Ahn^1,2^, Eun Hye Choi^1,3^, Hyunjung Lee^4,5^, Woochan Lee^4,5^, Jong-Il Kim^4,5^, Young Min Cho^1,3*^

^1^Department of Internal Medicine, Seoul National University College of Medicine, Seoul, Republic of Korea

^2^Department of Internal Medicine, Seoul National University Bundang Hospital, Seongnam, Republic of Korea

^3^Department of Internal Medicine, Seoul National University Hospital, Seoul, Republic of Korea

^4^Department of Biomedical Sciences, Seoul National University, Seoul, Republic of Korea.

^5^Genomic Medicine Institute, Medical Research Center, Seoul National University, Seoul, Republic of Korea

**Supplementary Table S1. Common DEGs of the liver, fat, and muscle in the VSG group compared to the sham-PF group**

|  | Liver (VSG vs. sham-PF) | | | Fat (VSG vs. sham-PF) | | | Muscle (VSG vs. sham-PF) | | |
| --- | --- | --- | --- | --- | --- | --- | --- | --- | --- |
| Gene | Log2 FC | Raw P value | Adj P value | Log2 FC | Raw P value | Adj P value | Log2 FC | Raw P value | Adj P value |
| Adgre4 | 1.984 | 0.005 | 0.111 | 1.520 | 0.023 | 0.673 | 1.850 | 0.005 | 0.474 |
| Aif1 | 1.874 | <0.001 | <0.001 | 1.046 | 0.012 | 0.478 | 1.437 | 0.014 | 0.800 |
| Aoah | 1.993 | <0.001 | 0.009 | 2.217 | 0.003 | 0.191 | 1.567 | 0.012 | 0.745 |
| Cd24a | 0.998 | 0.021 | 0.266 | 1.206 | 0.037 | 0.837 | -0.220 | 0.048 | 1.000 |
| Cd300e | 3.455 | <0.001 | 0.005 | 3.706 | <0.001 | 0.020 | 3.667 | <0.001 | NA |
| Cd300lf | 2.800 | <0.001 | <0.001 | 2.110 | 0.029 | 0.758 | 1.744 | 0.040 | NA |
| Clec4a1 | 2.989 | <0.001 | 0.013 | 1.469 | 0.012 | 0.467 | 1.088 | 0.001 | 0.221 |
| Cxcl13 | 4.590 | 0.004 | 0.103 | 3.869 | 0.002 | 0.172 | 3.826 | 0.003 | 0.386 |
| Dpep2 | 1.802 | 0.011 | 0.184 | -0.576 | 0.014 | 0.508 | 1.263 | 0.016 | 0.859 |
| Fgr | 2.526 | <0.001 | <0.001 | 1.554 | 0.016 | 0.549 | 1.837 | <0.001 | 0.164 |
| Gngt2 | 2.086 | <0.001 | <0.001 | 0.648 | 0.048 | 0.908 | 0.566 | 0.005 | 0.477 |
| H19 | 2.467 | 0.004 | 0.092 | -1.674 | <0.001 | 0.005 | 0.434 | <0.001 | <0.001 |
| Hbb-bs | 1.922 | <0.001 | <0.001 | 1.024 | 0.007 | 0.367 | 3.248 | <0.001 | 0.204 |
| Hbb-bt | 1.841 | <0.001 | <0.001 | 0.908 | 0.030 | 0.778 | 3.060 | 0.002 | 0.289 |
| Hspg2 | 0.876 | 0.016 | 0.226 | -0.632 | 0.039 | 0.860 | 0.322 | 0.016 | 0.857 |
| Ifngr1 | -0.777 | <0.001 | 0.023 | -0.305 | 0.046 | 0.907 | -0.299 | 0.004 | 0.450 |
| Ighm | 2.501 | <0.001 | 0.022 | 2.617 | 0.015 | 0.536 | 1.352 | <0.001 | 0.147 |
| Ighv11-2 | 5.925 | <0.001 | 0.011 | 3.710 | 0.014 | 0.511 | 6.094 | 0.045 | 1.000 |
| Ighv1-26 | 4.748 | 0.004 | 0.091 | 3.179 | 0.049 | 0.917 | 3.797 | 0.013 | NA |
| Igkc | 3.005 | <0.001 | <0.001 | 2.833 | 0.041 | 0.870 | 3.333 | 0.009 | 0.638 |
| Igkv1-110 | 2.627 | 0.010 | 0.179 | 3.774 | 0.012 | 0.482 | 5.089 | 0.023 | 0.991 |
| Igkv1-135 | 2.417 | 0.014 | 0.216 | 2.903 | 0.030 | 0.779 | 3.234 | 0.041 | NA |
| Iglc2 | 1.802 | 0.034 | 0.334 | 2.713 | <0.001 | 0.017 | 1.994 | 0.033 | NA |
| Il1b | 2.588 | <0.001 | 0.002 | 1.536 | 0.047 | 0.908 | 2.655 | 0.002 | 0.316 |
| Itgax | 2.413 | <0.001 | 0.009 | -1.457 | <0.001 | <0.001 | 1.043 | 0.009 | 0.631 |
| Jchain | 2.771 | <0.001 | 0.007 | 2.502 | 0.030 | 0.779 | 3.501 | <0.001 | 0.032 |
| Ltf | 9.214 | <0.001 | <0.001 | 3.232 | <0.001 | 0.020 | 3.341 | 0.022 | 0.974 |
| Marcksl1 | 1.226 | <0.001 | 0.005 | 1.257 | 0.038 | 0.851 | 0.616 | 0.049 | 1.000 |
| Mmp9 | 2.990 | <0.001 | 0.014 | 3.016 | <0.001 | 0.018 | 1.560 | 0.001 | 0.221 |
| Nfkbia | 0.737 | 0.011 | 0.193 | 0.728 | 0.010 | 0.426 | 0.347 | 0.043 | 1.000 |
| Ngp | 10.325 | <0.001 | <0.001 | 6.867 | <0.001 | 0.007 | 7.699 | 0.002 | 0.331 |
| Plxnd1 | 0.544 | 0.011 | 0.193 | -0.504 | 0.013 | 0.496 | 0.237 | 0.018 | 0.892 |
| Rgs2 | 1.575 | <0.001 | <0.001 | -0.640 | 0.011 | 0.453 | -0.390 | 0.010 | 0.661 |
| S100a9 | 5.456 | <0.001 | <0.001 | 4.351 | <0.001 | <0.001 | 3.951 | 0.003 | 0.392 |
| Siglece | 2.466 | <0.001 | 0.005 | 2.613 | 0.002 | 0.141 | 1.752 | 0.009 | 0.638 |
| Slc15a3 | 1.207 | <0.001 | 0.002 | -0.679 | 0.008 | 0.399 | 0.723 | 0.018 | 0.906 |
| Snca | 1.879 | 0.046 | 0.381 | 1.268 | 0.006 | 0.318 | 1.662 | <0.001 | 0.046 |
| Steap4 | 1.245 | 0.013 | 0.212 | 0.367 | 0.035 | 0.824 | 0.216 | 0.033 | 1.000 |
| Tmem176a | 0.733 | 0.013 | 0.205 | 0.948 | 0.048 | 0.908 | 0.595 | <0.001 | 0.205 |
| Tmem176b | 0.699 | 0.023 | 0.274 | 0.878 | 0.043 | 0.889 | 0.601 | <0.001 | 0.204 |

The criteria for DEG was raw P value < 0.05 in the liver, fat, and muscle. Log2 FC, log2 fold change; adj P value, adjusted P value

**Supplementary Figure S1. Flow cytometry analysis of the stromal vascular fraction isolated from the epididymal fat.**

The number of (a) total macrophages and the proportion of (b) M1 and (c) M2 macrophages. Lymphocyte population including (d) CD3^+^ T cells, (e) CD19^+^ B cells, and (f) CD3^+^/CD19^+^ ratio. Data are mean with SEM.

**Supplementary Figure S2. Pattern of the gene expression involved in metabolism pathways.**


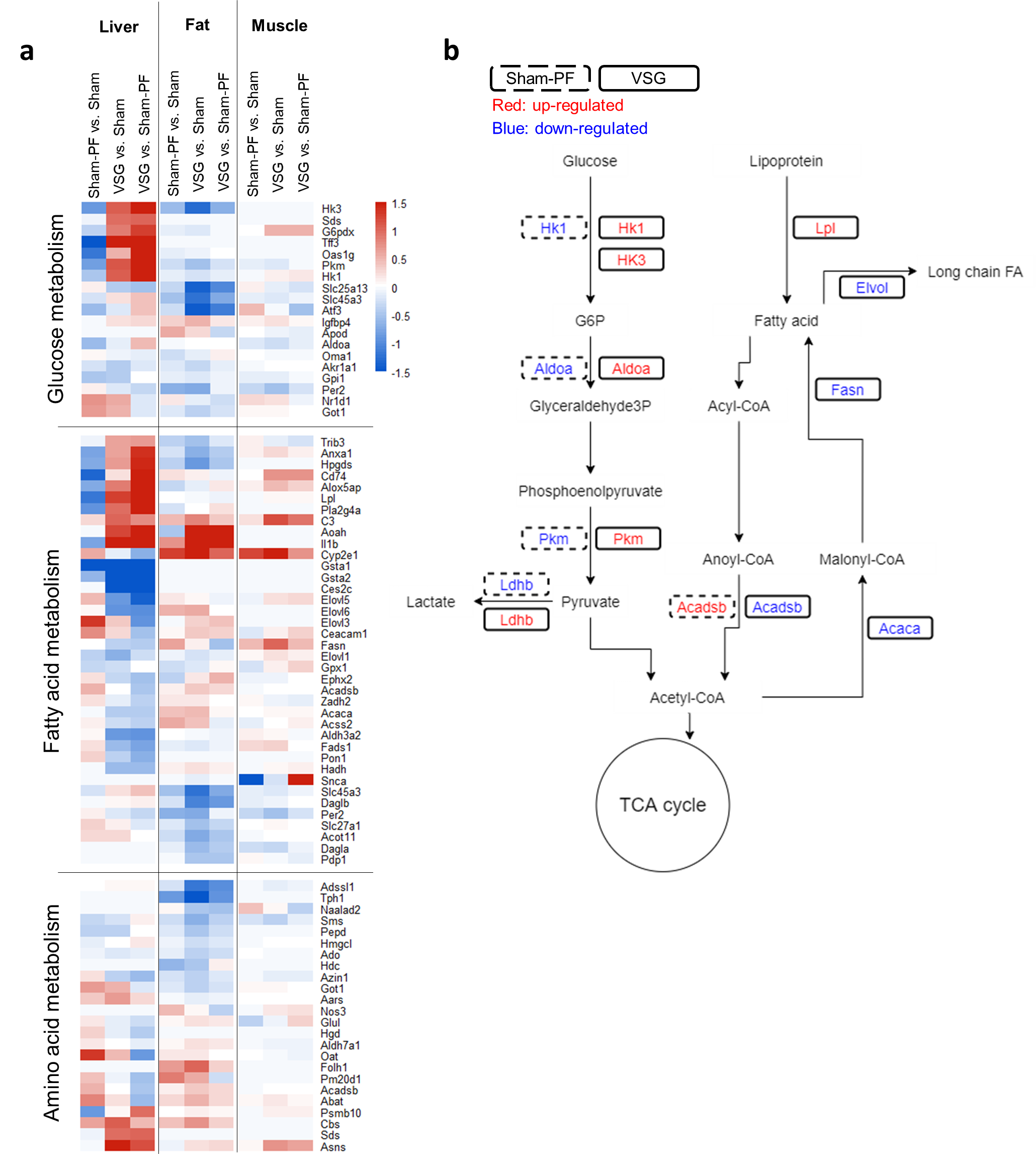


(a) The gene expression patterns of glucose metabolism, fatty acid metabolism, and amino acid metabolism in the liver, fat, and muscle. The heatmap shows log_2_ fold change. (b) The metabolic process of the liver changed in the VSG group (square) and the sham-PF group (dashed square). The metabolic pathway was modified from KEGG pathway (Kanehisa, M; Toward understanding the origin and evolution of cellular organisms. Protein Sci. 28, 1947-1951 (2019)).
